# Sex, Lies and fMRI—Gender Differences in Neural Basis of Deception

**DOI:** 10.1371/journal.pone.0043076

**Published:** 2012-08-29

**Authors:** Artur Marchewka, Katarzyna Jednorog, Marcel Falkiewicz, Wojciech Szeszkowski, Anna Grabowska, Iwona Szatkowska

**Affiliations:** 1 Department of Neurophysiology, Nencki Institute of Experimental Biology, Warsaw, Poland; 2 Faculty of Psychology, University of Warsaw, Warsaw, Poland; 3 Department of Radiology, Warsaw Medical University, Warsaw, Poland; 4 Warsaw School of Social Sciences and Humanities, Warsaw, Poland; University of Cambridge, United Kingdom

## Abstract

Deception has always been a part of human communication as it helps to promote self-presentation. Although both men and women are equally prone to try to manage their appearance, their strategies, motivation and eagerness may be different. Here, we asked if lying could be influenced by gender on both the behavioral and neural levels. To test whether the hypothesized gender differences in brain activity related to deceptive responses were caused by differential socialization in men and women, we administered the Gender Identity Inventory probing the participants’ subjective social sex role. In an fMRI session, participants were instructed either to lie or to tell the truth while answering a questionnaire focusing on general and personal information. Only for personal information, we found differences in neural responses during instructed deception in men and women. The women vs. men direct contrast revealed no significant differences in areas of activation, but men showed higher BOLD signal compared to women in the left middle frontal gyrus (MFG). Moreover, this effect remained unchanged when self-reported psychological gender was controlled for. Thus, our study showed that gender differences in the neural processes engaged during falsifying personal information might be independent from socialization.

## Introduction

The broadest definition describes deception as social behavior in which one person attempts to persuade another to accept as true what the deceiver believes to be untrue [1]. Majority of lies are motivated by self-presentation [2] and people tend to tell substantially more self-centered lies as opposed to other-oriented lies, most often lying about their emotions, actions, whereabouts, accomplishments, and knowledge [3].

One may question, however, the motivation behind lying and whether this motivation is the same for both sexes. Psychological studies claim that both men and women are equally prone to try to manage their appearance [2]. However, men are particularly likely to lie about their abilities and to exaggerate their personal characteristics and past experiences [4], [5], whereas women may lie more to promote intimacy, and their lies are intended to make other people feel better about themselves (e.g., [6]). A few studies attempted to test whether men lie equally often as women. Most studies found no gender differences in frequency of lying [3], [7]. Yet, when gender differences were found, it seems that women lied more often than men during non-anonymous conversations, but only when expecting future interactions [2]. However, when interactions are fully anonymous and deceptive messages can secure a monetary benefit, men are significantly more likely to lie than women [8].

Functional magnetic resonance imaging (fMRI) can allow researchers to link brain activity patterns directly to the cognitive or affective processes and behaviors they produce, including human deceptive behavior. Deception-related behavior was found to be associated with increased demands on the executive control system, such as allocating mental resources to processing task-relevant information (i.e., working memory – keeping truth in mind while lying), inhibitory control (i.e., suppressing truth), and guiding behavior in situations involving response conflict (i.e., task switching between truthful and deceptive responses; e.g. [9]; for reviews on neural correlates of deception see [10], [11]). Lie responses, in contrast to truthful responses, have been associated with increased activation in prefrontal regions linked to cognitive control, including the dorsolateral and ventrolateral prefrontal cortices and the anterior cingulate cortex [12], [13], [14], [15]. Interestingly, despite the wide range of different experimental paradigms that have been used (for detailed description see [16]), the pattern of activation of the frontal lobes, including the above-mentioned regions, associated with deception is indeed very similar.

From the neural perspective, lying thus seems to be a complex cognitive process and, as such, is likely to be influenced by gender, as a number of recent studies have shown sex differences in various cognitive tasks. Though numerous papers have revealed sex differences in behavioral studies (e.g., [17]), sex differences in brain activity during higher cognitive functions have only just begun to receive attention in the recent literature of functional neuroimaging. So far, a few studies have evaluated gender differences in healthy subjects regarding working memory tasks. Speck and collaborators [18] used fMRI to show that in a verbal working memory task, females performed more accurately (although slower) and had more left lateralized activations than men. Using a numerical working memory task, Bell, Willson, Wilman, Dave, & Silverstone [19] did not observe significant differences in the performance between the two genders, although the magnitude of the registered regional brain activation was larger in men. Greater regional brain activity in men when compared to women was found in the right superior frontal and inferior occipital gyrus and in the left inferior parietal gyrus.

Gender differences in inhibitory control, the other component of deception, have been researched more often. Studies have provided ample behavioral evidence for greater impulsivity in men than in women. For instance, men use illicit substances more frequently and in greater quantities than women [20], [21]; they also demonstrate greater sensation seeking and more frequent engagement in risk-taking behavior than women [22], [23]. Even among preschool children, a number of studies have found that girls were better than boys at avoiding forbidden objects, showing their greater compliance with adult requests (see [24] for meta-analysis). Importantly, however, even if no behavioral gender differences were observed in inhibitory control, as was demonstrated in a stop signal task, women and men differ in regional brain activation, which indicates that individuals may engage different brain regions and/or the same brain regions to different extents to achieve similar performance [25]. Compared to women, men recruited a larger number of brain regions during stop signal inhibitions. These structures included the bilateral medial frontal and cingulate cortices, the globus pallidus, the thalamus, and the parahippocampal gyrus. The authors suggested that men require more neural resources than women in inhibitory control, which might reflect their greater impulsivity.

We hypothesize that if women have an advantage in inhibitory control, as certain studies imply [20], [21], [22], [23], [24] and as is generally believed when applied to multitasking (task switching) [26], then they should show an advantage in deceptive behavior, which includes both of these processes. However, taking into consideration studies showing no behavioral but only brain activity differences between the sexes [25], we assumed that even if we did not find behavioral differences between men and women in lying as measured by reaction times or response rates, we would observe differences in regional brain activation, specifically in areas implicated in inhibitory control and task switching. To the best of our knowledge, the effect of gender differences in deception has never been directly studied using the functional brain imaging method. This was, therefore, the first aim of our study. To relate to most of the published studies on deception, we used an instructed lies paradigm, which, although suffers from some weaknesses ([27], [28]; see [16] for review), is still valued in the field [29], [30]) and constitutes a common interrogation tactics polygraph examiners use (for a review see: [31]).

Furthermore, it was demonstrated that brain regions which regulate cognitive control were more active during falsifying autobiographical information compared to nonautobiographical. Personal information is highly practiced and readily accessible, making it more difficult to suppress prepotent truthful responses [14]. Therefore, we added the additional dimension of personal (autobiographical) and general (nonautobiographical) question types to investigate sex differences when falsifying self-relevant and self-irrelevant information. As one experiences a greater amount of conflict and need for increased cognitive control when falsifying information about oneself rather than information of no personal significance, we predicted that gender-related differences in brain activity would be particularly pronounced during deception about personal information.

The second issue we tested was whether the postulated gender differences were a consequence of biological sex (and thus connected more to genetic code and evolutionary selection) or whether the differences were a result of an interaction between sex and various socialization factors, which may be reflected in psychological sex roles (e.g., [32]). The parental investment theory [33] claims that if we agree that the human genetic code still bears traces of the evolutionary forces it was subjected to, women’s advantage in inhibitory control might be seen as an evolutionary consequence. Another theory, called the differential socialization theory [34], postulates that in the process of socialization, women tend to be more concerned about the negative effects their behavior could have on others, whereas men portray themselves as more individualistic and risk-taking during socialization as part of the male gender role. To test whether the hypothesized gender differences in brain activity related to deceptive responses were associated with differential socialization in men and women, we administered an inventory probing the participants’ subjective social sex role. This was the second objective of this study.

## Results

### Behavioral Results

We analysed mean accuracy rates (AR) and reaction times (RT) using two separate repeated-measures ANOVAs with within-subject factors: Instruction (lie vs. tell the truth), Content (general vs. personal), and between-subjects factor: Gender (women vs. men).

**Figure 1:**
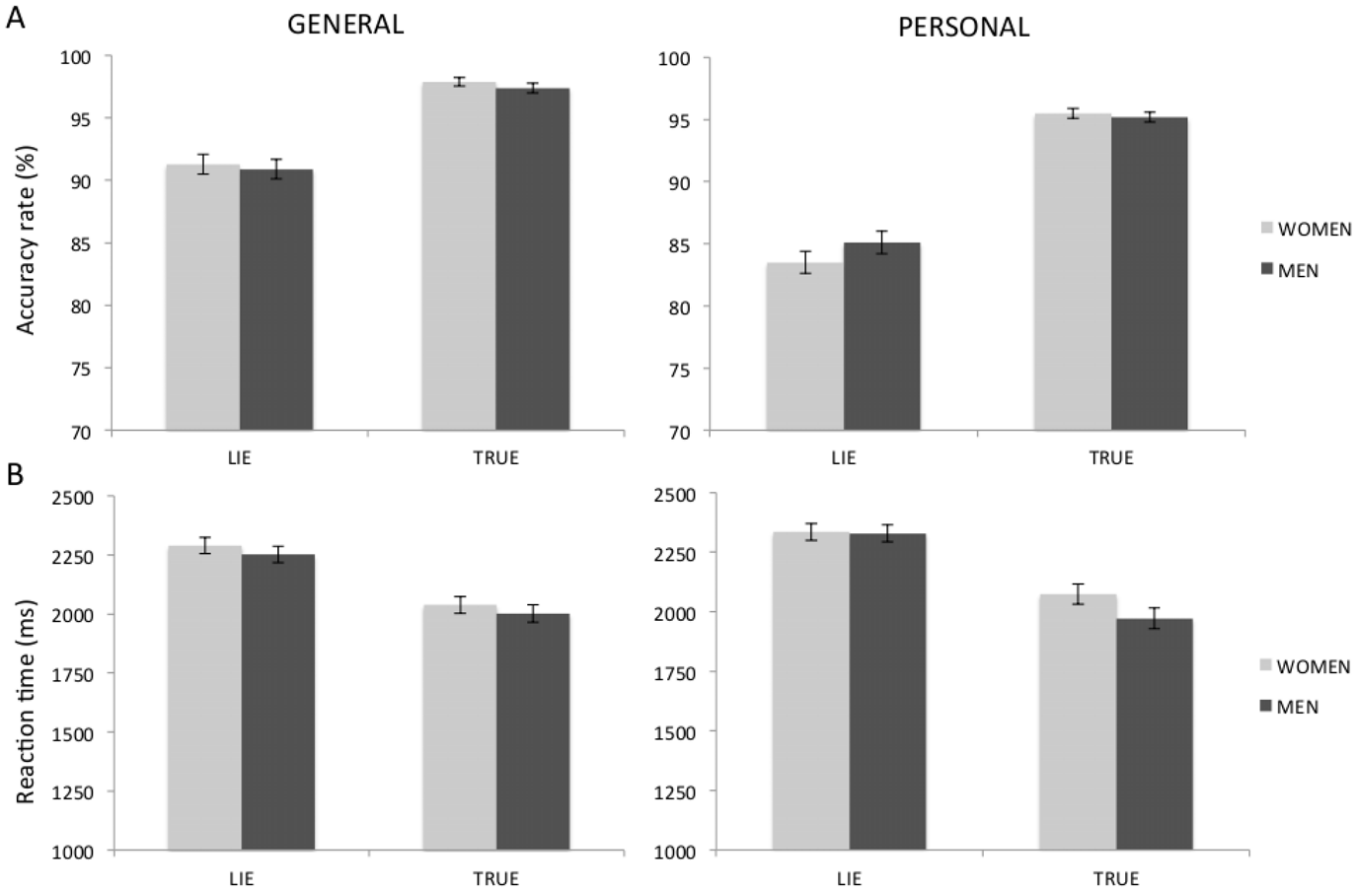
Behavioral results. Mean accuracy rates (A) and reaction times (B) of men and women for deceptive and truthful responses. Error bars represent standard error of the mean. No between-gender effect reached significance.

The analysis of AR revealed the main effect of Instruction (F(1,27) = 57.26; p<0.001) and Content (F(1,27) = 36.33; p<0.001) as well as the interaction of these two factors (F(1,27) = 7,94; p = 0.009). Subjects were less accurate when they had to lie (87.7%) in comparison to telling the truth (96.5%). They were also less accurate when replying to questions concerning personal (89.8%) compared to general information (94.4%). Neither Gender nor any interaction with this factor reached significance (see Figure 1A).

The analysis of RT revealed the main effect of Instruction (F(1,27) = 135.2; p<0.001) and Content (F(1,27) = 4.51; p = 0.043). Subjects answered significantly slower when they had to lie (2301 ms) in comparison to telling the truth (2021 ms). They were also slower when answering questions related to personal (2146 ms) compared to general (2177 ms) information. Again, neither Gender nor any interaction with this factor reached statistical significance (see Figure 1B).

**Figure 2:**
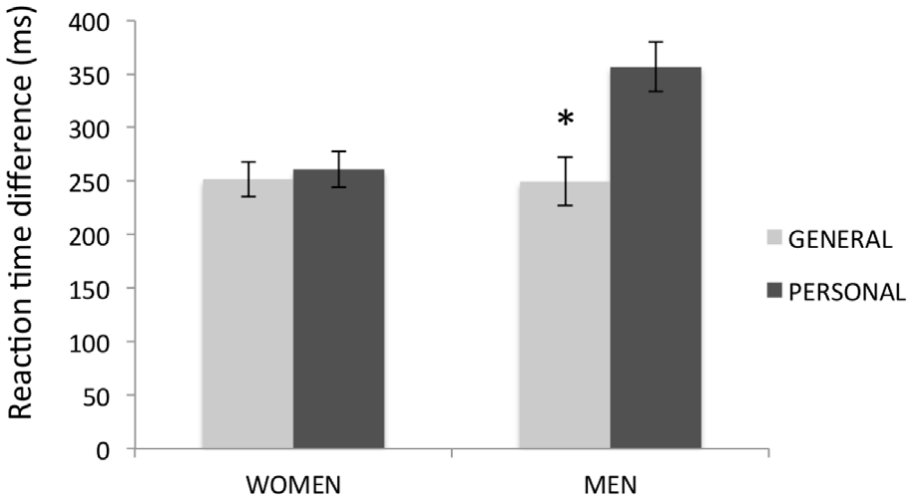
Differential reaction times (RT) of men and women for general and personal information. Error bars represent standard error of the mean. In men, differential RT for personal information were significantly longer than for general information, whereas in women no such differences were observed.

For RT additional analyses were conducted on differential RT i.e. RT when subjects’ had to lie minus RT when subjects’ had to tell the truth. We considered a within-subject factor of Content (general vs. personal) and between-subjects factor of Gender (women vs. men). Although the interaction Content×Gender did not reach significance (F(1,27) = 2.84; p = 0.103), in men differential RT for personal information were significantly longer (357 ms) than for general information (250 ms) (F(1,27) = 6.58; p = 0.016). No such differences were observed in women. However, differences between men and women in either personal or general content did not reach significance (see Figure 2).

### Imaging Results

#### Brain regions showing the main effect of deception

**Figure 3:**
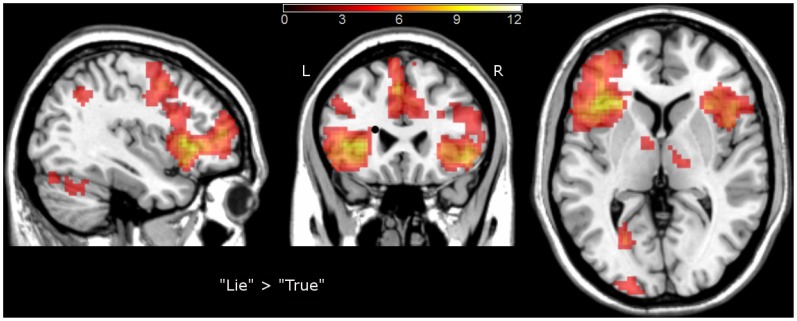
Brain regions showing increased activity during lying compared to truth-telling. The activations are superimposed on a Colin27 template image in the MNI space. The colored bar represents t-values; L – left side; R- right side.

In reference to previous studies showing brain areas involved in deception, we performed a “lie vs. truth” direct contrast comparison for general and personal information together. T-test contrast based analysis revealed brain regions involved in both lie conditions compared to the truth conditions: the insula bilaterally, the middle temporal gyrus (BA 21, 22) bilaterally; the left supplementary motor area (SMA, BA 6), the left occipital gyrus (BA 18), the left supramarginal gyrus (BA 40), the right inferior frontal gyrus (BA 47), the right middle frontal gyrus (BA10/46), and the right cerebellum (see Table 1 and Figure 3). Furthermore, three clusters were activated only during deception about general knowlegde: the right thalamus, the right middle frontal gyrus, and the left occipital gyrus. In contrast, lying about personal information led to activations in other brain regions: the right insula, the left caudate, the left thalamus, the right middle cingulate gyrus, the right postcentral gyrus and the precuneus bilaterally (see Table 2).

**Table 1:** Brain regions with significant BOLD activity for all subjects in the ‘lie vs. truth’ direct contrast for both general and personal information.

Brain Region	BA	MNI coordinates			Z-score	Clustersize
*All subjects*		x	y	z		
L Insula	13	−33	24	0	7.2	3980
L SMA	6	−3	18	51	6.6	
R Inferior Frontal Gyrus	47	48	21	−3	6.5	771
R Insula	13	39	18	−3	5.9	
R Cerebellum		30	−63	−27	5.7	2284
L Occipital Gyrus	18	−24	−102	0	5.7	
R Cerebellum		6	−57	−12	5.6	
R Middle Frontal Gyrus	10/46	36	51	9	4.7	132
L Middle Temporal Gyrus	21	−60	−39	−3	4.4	99
R Middle Temporal Gyrus	21	51	−36	−3	4.3	85
R Superior Frontal Gyrus	6	33	−6	63	4.1	34
L SupraMarginal Gyrus	40	−54	−51	27	4.0	67
L Middle Temporal Gyrus	22	−60	−54	18	3.7	59

All of the listed brain regions were cluster corrected at 10 contiguous voxels and met the significance threshold of p<0.05 (FWE). The x, y, z coordinates are the MNI coordinates. BA is the abbreviation for the approximate Brodmann’s areas; L is left; R is right; SMA is the supplementary motor area. Cluster size is the number of voxels activated in the regional cluster. Only the main peaks of activation within each cluster and their corresponding brain structures are reported.

**Table 2:** Brain regions with significant BOLD activity for all subjects in the ‘lie vs. truth’ direct contrast separately for general and personal information.

Brain Region	BA	MNI coordinates			Z-score	Clustersize
General Information		x	Y	z		
L Cerebellum		−3	−75	−27	5.92	170
R Cerebellum		9	−72	−36	3.83	
R Caudate		9	6	15	5.37	82
R Thalamus		15	−15	0	3.97	
L Inferior Frontal Gyrus	45/47	−39	27	0	5.18	1087
L Insula	13	−33	21	−9	4.70	
R Inferior Frontal Gyrus	46/47	48	21	0	5.09	404
R Middle Frontal Gyrus	45/46	33	15	36	4.48	
R SMA	6	6	12	57	4.96	503
L SMA	6	−3	18	51	4.66	
L Occipital Gyrus	17	−15	−96	−3	4.67	171
R Cerebellum		30	−63	−27	4.15	82
L Cerebellum		−30	−63	−30	4.03	59
**Personal Information**						
R Inferior Frontal Gyrus	46/47	36	33	0	5.72	424
R Insula	47	39	18	−6	5.31	
L SMA/Superior FrontalGyrus	6	−9	15	60	5.38	694
R SMA/Superior FrontalGyrus	6	9	9	63	5.07	
R Cerebellum		3	−63	−15	5.30	324
L Inferior Frontal Gyrus	45/47	−36	21	−9	5.14	783
L Insula	13	−30	24	3	5.08	
L Cerebellum		−33	−54	−33	4.69	67
L Caudate		−15	−6	21	4.38	63
L Thalamus		−12	−6	9	4.20	
R Caudate		15	−9	21	4.36	64
R Middle Cingulate Gyrus		21	−3	36	3.89	
L Precuneus	7	−6	−63	54	3.95	72
R Precuneus	7	6	−60	60	3.66	59
R Postcentral Gyrus	5	15	−48	72	3.44	

#### Brain regions differentiating self-relevant and self-irrelevant deceptive responses

**Figure 4:**
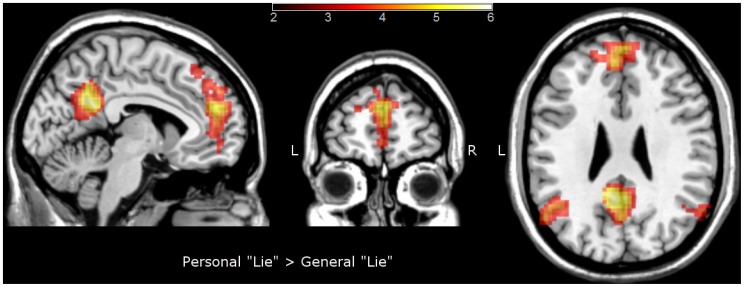
Brain activation map contrasting personal “lie” versus general “lie”. The activations are superimposed on a Colin27 template image in the MNI space. The colored bar represents t-values; L – left side; R- right side.

No brain regions were significantly active when comparing “general” vs “personal” within lie condition, whereas “personal” vs ”general” comparison revealed significant activations in the superior and medial frontal regions, the posterior cingulate, precuneus, middle temporal and angular gyri (see Table 3 and Figure 4). The interaction between content and gender, independently of response (lie or truth), produced no significant clusters of activity.

**Table 3:** Brain regions with significant BOLD activity for all subjects in the personal “lie” vs. general “lie” contrast.

Brain Region	BA	MNI coordinates			Z-score	Clustersize
*All subjects*		x	y	z		
L Precuneus	7	−3	−54	36	5.29	432
R Posterior Cingulate Cortex	31	3	−51	27	5.05	
L Superior Medial Gyrus	31	0	57	21	5.00	800
R Anterior Cingulate Cortex	10	12	48	12	4.61	
R Middle Temporal Gyrus	21	60	−9	−21	4.18	49
L Angular Gyrus	39	−54	−63	27	4.13	172
R Angular Gyrus	39	57	−63	30	4.12	70
R Middle Temporal Gyrus	45/46	57	−54	18	3.17	
L Middle Temporal Gyrus	45/46	−57	0	−18	3.86	60

#### Brain regions differentiating gender during deceptive responses

To address the question which brain structures are related to deceptive responses in the two sexes, we examined the contrasts: “lie vs. truth” separately in men and women focusing on general information, personal information, and both types of information together.

For both general and personal information, in the two sexes, significant increases in BOLD signal intensity were found in the right inferior frontal gyrus, bilateral caudate, the left thalamus and the right cerebellum. In women, significant activations were additionally found in the right insula and three frontal regions: the left superior frontal gyrus, the left superior medial gyrus, and the right middle frontal gyrus. In men, significant activations were also revealed in the left insula, the right thalamus, and in several frontal, parietal, temporal, and occipital cortical areas including the superior frontal gyrus and the precuneus in the right hemisphere, as well as the middle occipital gyrus, the middle temporal gyrus, the parahippocampal gyrus, the supramarginal gyrus and the precuneus in the left hemisphere (see Table 4).

**Table 4:** Brain regions with significant BOLD activity for the ‘lie vs. truth’ direct contrast for both general and personal information separately in men and women groups.

*Brain Region*	*BA*	*MNI coordinates*			*Z-score*	*Cluster* *size*
*Men*		*x*	*y*	*z*		
R Superior Frontal Gyrus	6	3	12	60	6.21	2606
L Inferior Frontal Gyrus	47	−39	27	0	5.92	
R Cerebelum		33	−57	−33	6.00	1868
Left Middle Occipital Gyrus		−18	−96	0	5.31	
R Inferior Frontal Gyrus	47	39	18	−3	5.49	406
L Middle Temporal Gyrus	21	−57	−39	−6	4.73	100
L Parahippocampal Gyrus	19	−39	−45	−3	3.21	
R Thalamus		15	−12	15	4.52	46
R Caudate		21	−15	27	3.72	
Left SupraMarginal Gyrus	40	−54	−48	27	4.37	92
L Precuneus	7	−6	−69	48	4.33	60
R Precuneus	7	6	−75	57	3.90	
L Caudate		−15	−6	21	4.13	47
L Insula		−30	−6	24	3.54	
L Thalamus		−3	−12	15	4.27	72
R Thalamus		3	−18	12	4.26	
**Women**						
**R Insula**		−33	24	0	5.25	368
R Inferior Frontal Gyrus	47	33	30	0	5.22	209
R Caudate		15	−9	21	4.57	84
L Superior Frontal Gyrus	6	−9	21	60	4.41	296
L Superior Medial Gyrus	8	−3	18	51	4.23	
L Thalamus		−12	−3	12	4.36	61
L Caudate		−15	3	18	4.14	
R Cerebelum		3	−60	−9	4.15	52
R Middle Frontal Gyrus	46	48	27	27	3.99	73

For general information, several brain regions were activated in both men and women: the left inferior frontal gyrus, the left insula, the left SMA, the left inferior frontal gyrus, the left superior medial gyrus, and the right cerebellum. Four additional regions were activated in women: the right SMA, the right inferior frontal gyrus, the left occipital gyrus, and the left cerebellum. In men, significant activations were additionally found in four brain areas: the left precentral gyrus, the left middle frontal gyrus, the left middle occipital gyrus, and the right insula. To directly analyze the gender differences in brain activity regarding deception, we compared the contrast “lie vs. truth” between men and women. Neither the comparison of “women vs. men” nor the comparison of “men vs. women” revealed significant suprathreshold clusters (see Table 5).

**Table 5:** Brain regions with significant BOLD activity for men and women separately in the ‘lie vs. truth’ direct contrasts for general information.

*Brain Region*	*BA*	*MNI coordinates*			*Z-score*	*Cluster* *size*
*Men*		*X*	*Y*	*z*		
L Insula	13	−36	21	3	5.6	582.0
L Inferior Fronatal Gyrus	44	−42	12	9	4.4	
L SMA	6	0	12	57	4.3	209.0
L Cingulate Cortex/Superior Fronatl Gyrus	32	−3	30	33	4.3	
L Superior Medial Gyrus/Superior Fronatl Gyrus	32	−9	21	42	4.1	
R Insula	13	42	18	0	4.3	74.0
L Precentral Gyrus	6	−36	0	51	4.2	119.0
R Cerebellum		33	−54	−33	4.2	68.0
L Middle Frontal Gyrus	45/46	−45	18	36	4.0	64.0
L Inferior Frontal Gyrus	46/47	−45	6	24	3.7	
L Middle Occipital Gyrus	18	−27	−99	−3	3.7	42.0
**Women**						
R Cerebellum		−3	−75	−27	4.78	80
L Cerebellum		−15	−72	−33	4.38	
R SMA	6	9	12	66	4.74	82
L SMA	6	−3	18	60	4.07	
L Inferior Fronatal Gyrus		−39	27	−3	3.37	
L Insula	13	−30	18	−12	3.25	
L Occipital Gyrus	46/47	−12	−96	−6	4.15	60
R Inferior Frontal Gyrus	45/46	48	27	30	4.09	70
L Superior Medial Gyrus	8	−3	27	45	4.01	44
R Cingulate Cortex	32	6	21	39	3.14	
L Inferior Frontal Gyrus	45/46	−39	12	24	3.87	57

**Figure 5:**
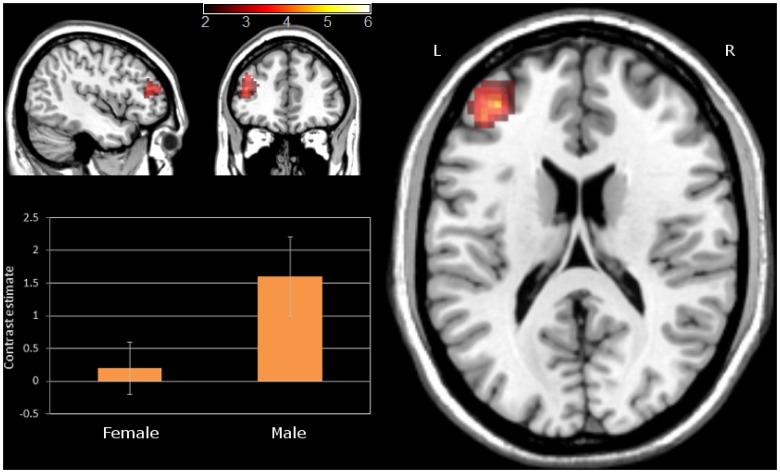
Differences between lying and truth-telling in men and women in the left middle frontal gyrus area. The bar chart represents the mean contrast value of the cluster. The activation is superimposed on a Colin27 template image in the MNI space. The colored bar represents t-values; L – left side; R- right side.

For personal information, **t**he analysis in women showed increases in BOLD signal in two clusters comprising the bilateral inferior frontal gyri and the right insula. The same regions were revealed in men; however, for men, significant activation was also found in broad parts of the prefrontal cortex extending to the middle frontal gyrus and to the SMA and also subcortically in the caudate nucleus, the thalamus and the cerebellum (see Table 6). Direct comparison of “women vs. men” revealed no significant suprathreshold clusters. However, men vs. women direct contrast showed significantly increased activation in men in one cluster of 40 voxels in the left middle frontal gyrus (MFG) with two local maxima peaks (x = −42, y = 39, z = 21, Z = 3.85 and x = −35, y = 42, z = 15, Z = 3.51; see Figure 5). To explore the influence of psychological gender on deception, we extracted the signal from the left MFG cluster using MarsBaR software [35] and ran additional correlation analyses in SPSS v.18 with the scores of masculinity and femininity from the Gender Identity Inventory. These analyses revealed that neither masculinity, femininity nor the difference femininity-masculinity (in men, women and in both groups together) significantly correlated with the differences in brain activity of the left MFG.

**Table 6:** Brain regions with significant BOLD activity for men and women separately in the ‘lie vs. truth’ direct contrasts for personal information.

*Brain Region*	*BA*	*MNI coordinates*			*Z-score*	*Cluster* *size*
*Men*		*X*	*Y*	*Z*		
R SMA/Superior Frontal Gyrus	6	9	9	63	5.14	561
L SMA/Superior Frontal Gyrus	6	−6	12	51	4.75	
L Middle Frontal Gyrus	45/46	−36	39	18	5.08	721
L Inferior Frontal Gyrus	45	−51	15	3	4.89	
R Inferior Frontal Gyrus	47	39	33	0	5.06	291
R Insula	13	39	18	−3	4.72	
R Cerebellum		6	−5	−12	4.97	583
L Caudate		−21	−9	27	4.80	36
R Middle Frontal Gyrus	46	30	39	21	4.60	70
L Superior Frontal Gyrus	6	−27	−3	66	3.97	57
L Precentral Gyrus	6	−36	−3	66	3.47	
L Thalamus		−3	−2	12	3.88	38
L Calcarine Sulcus	17	−12	−7	9	3.51	44
**Women**						
R Inferior Frontal Gyrus	47	36	33	0	4.38	55
R Insula	13	27	24	−3	4.14	
L Inferior Frontal Gyrus	47/45	−36	24	−12	3.99	109

## Discussion

In the present study, we aimed to investigate whether men and women differ when falsifying general (self-irrelevant) and personal (self-relevant) information. As for the behavioral performance, men and women did not differ when it came to accuracy or RTs. The only difference was observed in differential RTs (i.e., the differences between lying and truth telling). In men, differential RTs for personal information were significantly longer than for general information. No such differences were observed in women. This may potentially suggest that for men lying about personal information is more difficult and produces a more significant interference effect than does lying about general information, whereas for women both types of lying have similar levels of difficulty. In agreement with this hypothesis, we found differences in neural correlates underlying deceptive responses between men and women significant only in case of personal information.

In line with previous studies, both groups showed deception-related activations in a number of regions. We found both common and unique neural correlates that underlied falsifying self-irrelevant and self-relevant information. A functional overlap between these two types of lying (including the bilateral prefrontal areas of the inferior frontal gyrus and the superior frontal gyrus/supplementary motor area, as well as bilateral cerebellum, the left insula and the right caudate) bears resemblance to brain areas that generally contribute to executive control (e.g. [36], [37]) and is in line with the concept of deception being an executive control task. Particularly, prefrontal cortex involvement is in clear agreement with previous neuroimaging studies that have shown its predominant role in deception [13], [15], [38]. Recently, Karim et al. [39] provided evidence for a causal connection between anterior prefrontal cortex activity and deceptive behavior. Cathodal transcranial direct current stimulation, which results in the suppression of cortical excitability, significantly improved lying (i.e., reduced reaction times and decreased the sympathetic skin-conductance response and guilty feeling while deceiving the interrogator) when applied to the anterior prefrontal region. The anterior insula appeared to be involved in processing information that may have consequences for subjects [40] and may also be an integral hub in mediating dynamic interactions between other large-scale brain networks involved in externally oriented attention and internally oriented or self-related cognition [41]. The cerebellum has been implicated in deception (e.g., [13]) due to the role it plays in episodic memory retrieval [42] and, more generally, in verbal working memory [43]. The caudate nucleus was initially thought to be primarily involved in controlling voluntary movement. More recently, however, it has been also implicated in cognitive control [44], particularly in task switching [45]. Unique activations for each deception type were also delineated, showing that type of lie modulates patterns of brain activation related to deception. Consistently with previous study [14], falsifying autobiographical responses produced more robust neural effects relative to falsifying non-autobiographical responses, again proving that the amount of conflict induced and cognitive control needed is much greater when dealing with personal information. Next, these two processes were examined for each gender.

In men and women falsifying non-autobiographical responses recruited similar brain areas. A direct between group contrast revealed no areas of activity that would differ between men and women. In contrast, when the two sexes were directly compared for activations during falsifying autobiographical information, women revealed no areas of significantly higher activation than men, whereas men showed higher BOLD signal compared to women in the left middle frontal gyrus (MFG). Changes in the activity of the left MFG were shown to be associated with the generation of deceptive responses in healthy individuals [46] and with an impairment of the ability to make deceptive responses in patients with Parkinson’s disease [47], which indicates the importance of the left MFG for deceptive behavior. The left MFG has also been linked to inhibitory mechanisms [48], [49], [50]. Patients with lesions restricted to the left middle and inferior frontal gyri demonstrated profound deficits in resolving interference from previous items in working memory [50]. These prefrontal regions thus seem to subserve a general, nonmnemonic function of selecting relevant information in the face of competing alternatives. Lying could be considered an action composed of the simultaneous monitoring and inhibition of truthful responses, switching attention between truthful and false responses, and selecting the false response. Thus, sex-related differences in the activation of the left MFG observed in the present study may reflect distinct efficiency in selecting self-relevant information in men and women. Based on the idea that the amount of neural activity depends on the computational demand that the task imposes [51], one possible explanation of the observed higher intensity of the left MFG activation in men can be considered as indicative of a greater effort. It may also suggest that mechanisms of selecting relevant information in the pursuit of a higher behavioral goal are less efficient in men. Similarly, in a stop-signal inhibition task, men required more neural resources to inhibit a pre-potent motor response [25]. This points to inhibition as another component of deception, in addition to the selection of relevant information. Further studies should work to identify which of the cognitive processes involved in deception (working memory, inhibition or task switching) might explain the gender differences observed here. Interestingly, in the present study, gender differences in neural correlates of deception were observed only in case of self-relevant, personal information. It has been previously demonstrated and observed also in this study that personal relevance noticeably influences patterns of behavioral and neural activity within the context of deceptive behavior. It is conceivable that only when falsifying personal information, the amount of conflict and cognitive control needed is strong enough and more sensitive to reveal gender differences.

Finally, we examined the relationship between the sex related brain activity differences and subjective scores of masculinity and femininity from the Gender Identity Inventory and found no such association. If we assume that the Gender Identity Inventory measures how well one can fit into the traditional gender roles, the sex differences in brain activation as found in the present study are unlikely to be influenced by differential socialization in men and women.

In conclusion, to our knowledge, this is the first fMRI study that reports gender differences in the neural correlates of deception. We provide evidence that despite comparable performance and brain activity on the deception task related to general information, there are sex differences in brain activity when falsifying personal information. We observed overall enhanced activation in men, particularly in the left middle frontal gyrus. This may suggest that men found the personal deception task more difficult to perform, probably due to less efficient mechanisms for selecting relevant information in the pursuit of a behavioral goal. Therefore, the results support the idea of studying men and women as distinct groups in functional imaging studies on deception. However, the interpretation of this study is limited by the low ecological validity of the experimental paradigm. The participants were instructed to make deceptive responses, which may not be equivalent to deception during real life conditions or even in computerized games (see [52]). Artificial settings in the instructed lies paradigm, in comparison to real life situations, precludes the voluntary intention to deceive and does not evoke emotional involvement, both of which are crucial components of deception. Finally, deception in the real world is far more complex and sophisticated than the deception in our study. Therefore, further studies should verify if the different patterns of activation in men and women reported here are also present in genuinely deceptive tasks.

## Materials and Methods

### Ethics Statement

The Bioethics Committee of Warsaw Medical University approved the experimental protocol, and informed written consent was obtained from all subjects prior to the study.

### Participants

Twenty-nine right-handed and healthy volunteers (15 female and 14 male) between the ages of 21 and 28 participated in this study (female mean age = 23.7; SD = 2, male mean age = 24.9; SD = 2.3). Study groups were balanced in years and type of education (female mean years of education = 16.07; SD = 1.64, male mean years of education = 16.73; SD = 1.85; mostly biology and psychology students or graduates). Right-handedness was confirmed using the Edinburgh Inventory [53]. The participants were PhD students, MSc students or employees of the Nencki Institute of Experimental Biology, Warsaw, Poland. Participants had no history of psychiatric or neurological illness, either past or present. Subjects were paid PLN 100 (approximately 25 Euros) for their participation. All participants filled out the Gender Identity Inventory [54], which was created on the basis of Sandra Bem’s Sex Role Inventory [55] and was specifically adjusted to the Polish population.

### Stimuli and Task

All subjects underwent the same experimental procedure. First, they completed a paper-pencil questionnaire concerning personal information and general knowledge. On the following day, they underwent an fMRI scan during which they answered questions that were prepared based on the information obtained from the questionnaire. There were 120 questions in total: 60 personal and 60 general. Out of each 60 questions 30 were presented with the instruction to give a false reply and 30 with the instruction to tell the truth. Two types of answers were possible for each category of questions: 15 were supposed to be answered with a “Yes” and 15 with a “No”. All experimental conditions were fully counterbalanced with respect to the instruction, type of question and type of answer. The questions were presented in a pseudo random order identical for every subject. The questions were kept as simple and as short as possible. The average length (5.2 words) was also matched across conditions.

**Figure 6:**
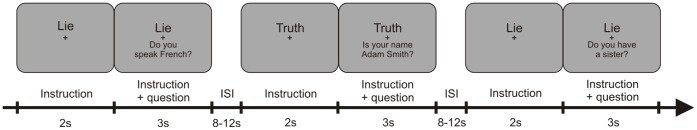
Experimental design of the study. The instruction was presented above the fixation point and, after 2 seconds, was followed by a question that appeared below the fixation point for 3 seconds. The inter-stimulus interval was varied from 8–12 s (for details see text).

Each trial started with a centrally presented fixation point with the instruction (to lie or to tell the truth) being presented above the fixation which, after 2 seconds, was followed by a question being displayed for 3 seconds at the center of the fixation point. The instruction was displayed throughout the entire duration of the trial (Figure 6). The inter-stimulus interval varied from 8 to 12 seconds. Subjects gave answers using a 2-button response pad; the meaning of the buttons was changed for half of the participants. Accuracy and reaction times were recorded.

As the motivation to lie is rather low in laboratory studies, we sought to increase the subjects’ motivation as follows: (1) Our recent studies have shown that monetary rewards motivated healthy subjects to perform better [56] and influenced the effective connectivity between brain regions supporting motivation-cognition interaction [57] on tasks that required cognitive control to perform at an optimal level. As deception is an example of such a task, in the present study, a monetary compensation for correct performance was introduced. (2) Subjects were told that during the fMRI session a lie expert would observe their facial expressions on a camera hidden inside the MRI coil (however, no camera was installed) and would evaluate the changes of their galvanic skin response (a fake device had been attached to the middle finger of their left hand). Those judged “the best liars and truth-tellers” would win a financial reward of approximately 40 Euros. The actual assessment was based on the accuracy rates and reaction times.

### Image Acquisition and Data Analysis

Whole brain imaging was performed with a 1.5-Tesla MRI scanner (Magnetom Avanto; Siemens, Erlangen, Germany) equipped with 32-channel phased array head coil. Head movements were minimized with cushions placed around the participants’ heads. A T2*-weighted echo planar imaging (EPI) sequence was used for functional imaging with the following parameters: time repetition = 2000 ms; time echo = 50 ms; flip angle = 90 deg; inplane resolution = 2.5×2.5 mm; field of view = 240 mm; and 23 axial slices, with 6 mm slice thickness and no gap between slices. For each subject, the functional run consisted of 915 volumes lasting 30 minutes and 30 seconds. Detailed anatomical data of the brain were acquired with sagittal T1-weighted (time repetition = 1720 ms; time echo = 2.92 ms) and T2-weighted (TR = 3200 ms; TE = 381 ms) MPRAGE sequences with isotropic voxel size (1×1×1 mm).

Statistical Parametric Mapping (SPM8, Wellcome Trust Center for Neuroimaging, London, UK) running on MATLAB 7.9 (The Math-Works Inc. Natick, MA, USA) was used for data processing and statistical analyses. Images were corrected for head movement (spatial realignment). Slice acquisition time was corrected by taking the middle slice in time as a reference. Both anatomical scans were coregistered with the mean of realigned functional images. The upgraded implementation of unified segmentation (“New Segment”) was used to segment anatomical images into grey matter, white matter and other tissues. Data from both the T1- and T2-weighted scans of the same subject were used to obtain more accurate results. High-dimensional Diffeomorphic Anatomical Registration Through Exponentiated Lie Algebra (DARTEL, [58]) was used to create a group-specific template and flow fields (containing mappings between the subject-specific image and the template). This approach was applied to minimize the influence of gender differences on the brain structure. The template was affine registered with Montreal Neurological Institute (MNI) space. The functional images were normalized using compositions of flow fields and template affine transformation parameters and were resampled to a 3 mm isotropic voxel size. Finally, the normalized functional images were smoothed with a 6 mm isotropic Gaussian kernel.

In the first-level statistical analysis, experimental stimuli were split into separate regressors based on the instruction-answer scheme. Misses and incorrect responses were entered as a separate regressor and excluded from further analysis. Head movement parameters were also entered as covariates into the design matrix. Each stimulus was modeled as an event of 3 s duration, starting when the question was presented and ending when it disappeared from the screen. All stimulus functions were convolved with the canonical HRF basis function. In a second-level group random effects analysis, linear contrasts of the parameter estimates were subjected to one-sample t-tests. Anatomical labels were assigned to functional activations using a probabilistic cytoarchitectonic map [59], [60]. All the reported data were family-wise error corrected (FWE) for multiple comparisons at the cluster level, and a significance threshold of p<0.05 was applied (cluster size>10 voxels). Only the main peaks of activation with a Z-score within each cluster and their corresponding brain structures were reported. The number of voxels activated in significant clusters is presented in the tables.
